# Supplementary material for: Novel Driver Strength Index highlights important cancer genes in TCGA PanCanAtlas patients
Source: PeerJ. 2022 Aug 11;10:e13860. doi: 10.7717/peerj.13860 (PMC9375969; doi:10.7717/peerj.13860)
Supplement: Supplemental Information 2 [file peerj-10-13860-s002.zip › COHORTS/genes plots/age/2021_8_16_14_9_distribution_cohorts__25.pdf]

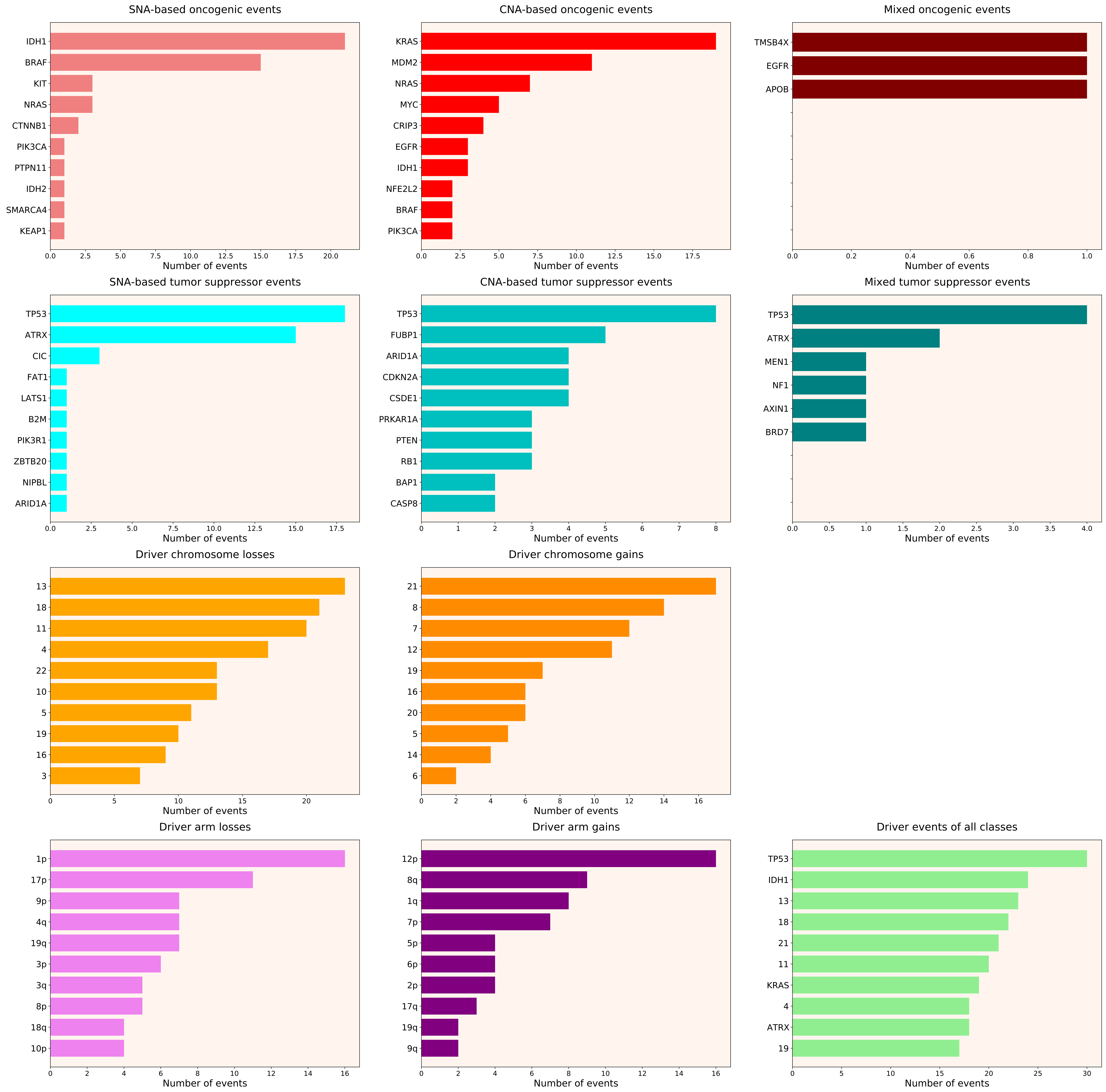
SNA-based oncogenic events
IDH1
BRAF
KIT
NRAS
CTNNB1
PIK3CA
PTPN11
IDH2
SMARCA4
KEAP1
0.0 2.5 5.0 7.5 10.0 12.5 15.0 17.5 20.0
Number of events
CNA-based oncogenic events
KRAS
MDM2
NRAS
MYC
CRIP3
EGFR
IDH1
NFE2L2
BRAF
PIK3CA
0.0 2.5 5.0 7.5 10.0 12.5 15.0 17.5
Number of events
Mixed oncogenic events
TMSB4X
EGFR
APOB
0.0 0.2 0.4 0.6 0.8 1.0
Number of events
SNA-based tumor suppressor events
TP53
ATRX
CIC
FAT1
LATS1
B2M
PIK3R1
ZBTB20
NIPBL
ARID1A
0.0 2.5 5.0 7.5 10.0 12.5 15.0 17.5
Number of events
CNA-based tumor suppressor events
TP53
FUBP1
ARID1A
CDKN2A
CSDE1
PRKAR1A
PTEN
RB1
BAP1
CASP8
0 1 2 3 4 5 6 7 8
Number of events
Mixed tumor suppressor events
TP53
ATRX
MEN1
NF1
AXIN1
BRD7
0.0 0.5 1.0 1.5 2.0 2.5 3.0 3.5 4.0
Number of events
Driver chromosome losses
13
18
11
4
22
10
5
19
16
3
0 5 10 15 20
Number of events
Driver chromosome gains
21
8
7
12
19
16
20
5
14
6
0 2 4 6 8 10 12 14 16
Number of events
Driver arm losses
1p
17p
9p
4q
19q
3p
3q
8p
18q
10p
0 2 4 6 8 10 12 14 16
Number of events
Driver arm gains
12p
8q
1q
7p
5p
6p
2p
17q
19q
9q
0 2 4 6 8 10 12 14 16
Number of events
Driver events of all classes
TP53
IDH1
13
18
21
11
KRAS
4
ATRX
19
0 5 10 15 20 25 30
Number of events
